# Crystal structures of two unusual, high oxidation state, 16-electron irida­benzenes

**DOI:** 10.1107/S2056989015018952

**Published:** 2015-10-14

**Authors:** Daniel T. Chase, Lev N. Zakharov, Michael M. Haley

**Affiliations:** a Department of Chemistry & Biochemistry and Materials Science Institute, University of Oregon, Eugene, Oregon 97403-1253, USA; b CAMCOR, University of Oregon, 1443 East 13th Avenue, Eugene, Oregon 97403, USA

**Keywords:** Irida­benzene, metalla­benzene, aromaticity, oxidation state, coordinatively unsaturated, crystal structure

## Abstract

Treatment of carbon­yl(1,2-diphenylpenta-1,3-dien-1-yl-5-yl­idene)bis­(tri­phenyl­phosphane)iridium, [IrCO(—C(Ph)=C(Ph)—CH=CH—CH=)(PPh_3_)_2_], with either bromine or iodine produced di­bromido­(1,2-diphenylpenta-1,3-dien-1-yl-5-yl­idene)(tri­phenyl­phosphine)iridium(III), [IrBr_2_{—C(Ph)=C(Ph)—CH=CH—CH=}(PPh_3_)], (I), and (1,2-diphenylpenta-1,3-dien-1-yl-5-yl­idene)di­iodido­(tri­phenyl­phosphane)iridium(III), [IrI_2_{—C(Ph)=C(Ph)—CH=CH—CH=}(PPh_3_)], (II), respectively, which are two rare examples of 16-electron metalla­benzenes. Structural elucidation of (I) and (II) reveals that these isotypic irida­benzenes are unusual, not only in their electron count, but also in their coordination sphere of the Ir^III^ atom where they contain an apparent open coordination site. The crystal structures of (I) and (II) confirm that the mol­ecules are complexes containing five-coordinated Ir^III^ with only one tri­phenyl­phosphine group bound to the iridium atom, unambiguously proving that the mol­ecules are indeed 16-electron, high-oxidation-state irida­benzenes. The coordination geometry of the Ir^III^ atom in both structures can be best described as a distorted square pyramid with the P, two Br (or I) and one C atom in the basal plane and another C atom in the apical position.

## Abstract 2

Treatment of an irida­benzene with either bromine or iodine generates high-oxidation-state Ir^III^ irida­benzenes that contain an open coordination site.

## Chemical context   

Metalla­benzenes are a rare class of organometallic compounds in which a CH unit is isolobally substituted with a transition metal fragment (Bleeke, 2001 ▸; Wright, 2006 ▸). Postulated in a seminal paper in 1979 (Thorn & Hoffmann, 1979 ▸), metalla­benzenes have been shown to be feasible through numerous synthetic methodologies and now claim residence in the third and second row transition metals. Our research has focused on the synthesis and properties of metalla­benzenes and their valence isomers using 3-vinyl-1-cyclo­propenes as the source for the five-carbon backbone (Landorf & Haley, 2006 ▸). In certain instances, the metalla­benzenes can undergo reductive elimination to afford η^5^-Cp complexes (Wu *et al.*, 2007 ▸). Although such a pathway has potential synthetic utility, for our studies this represents a deleterious side reaction that hinders an effective, detailed examination of metalla­benzenes. Computational work by van der Boom and coworkers suggests that metalla­benzenes containing metal atoms with higher oxidation states may be resistant toward the reductive elimination pathway (Iron *et al.*, 2003 ▸). This prediction inter­ested us as prior studies have shown that Ir^I^ irida­benzenes can be readily oxidized with Ag^I^ salts or halogens to generate high oxidation state Ir^III^ irida­benzenes; hence, we sought to synthesize neutral irida­benzenes of higher oxidation state as initially demonstrated by Bleeke and coworkers (Bleeke *et al.*, 1997 ▸). Herein we report the synthesis and structures of irida­benzenes (I) and (II), two rare examples of high oxidation yet coordinatively unsaturated 16-electron Ir^III^ irida­benzenes.
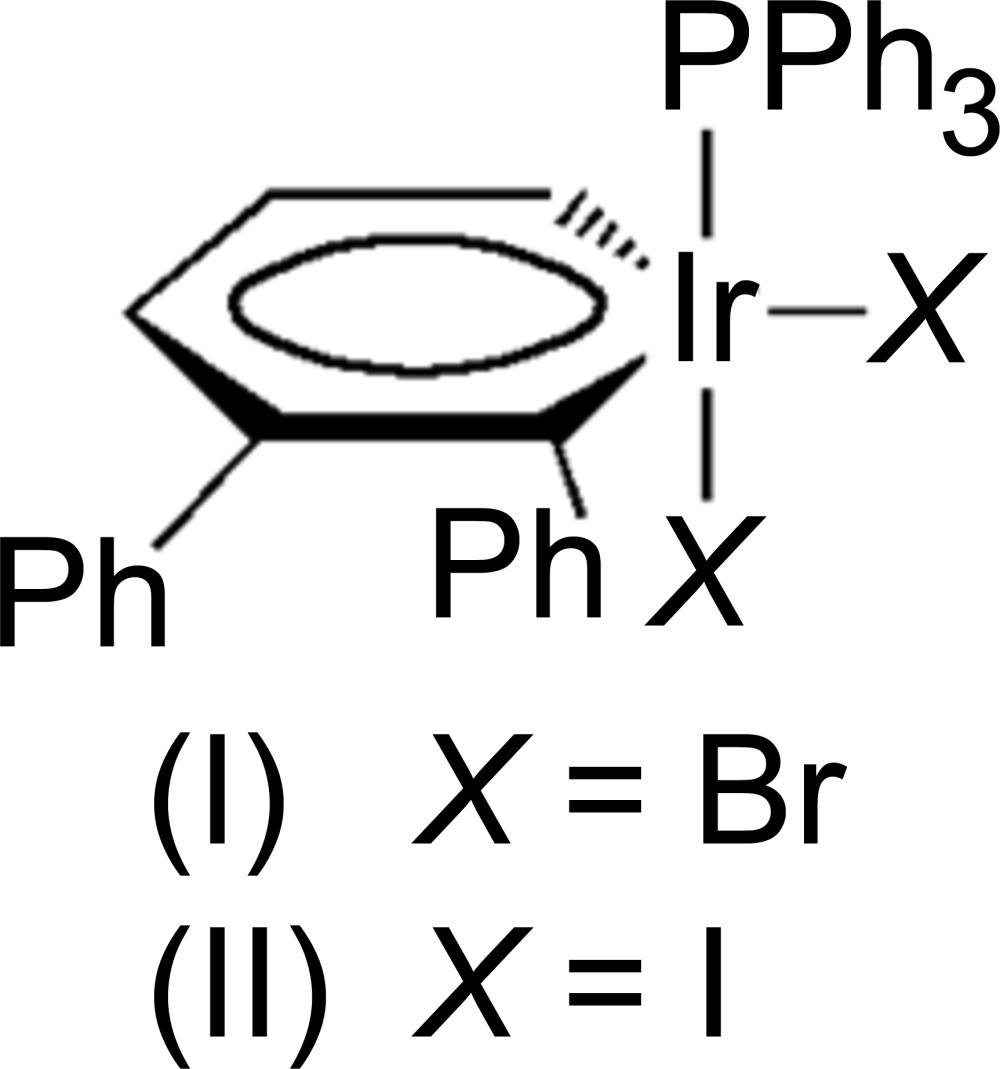


## Structural commentary   

**Figure 1:**
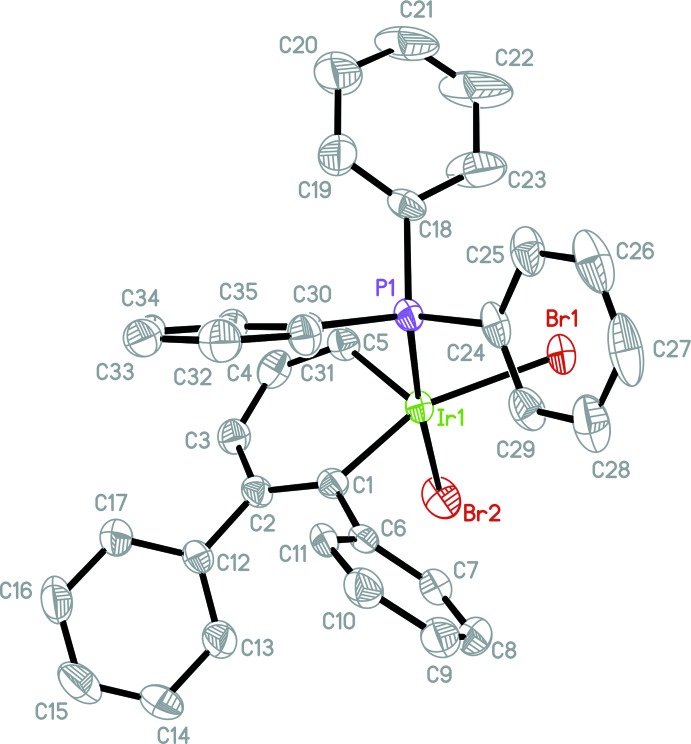
The mol­ecular structure of (I), showing the atom-labelling scheme. Displacement ellipsoids are drawn at the 50% probability level.

**Figure 2:**
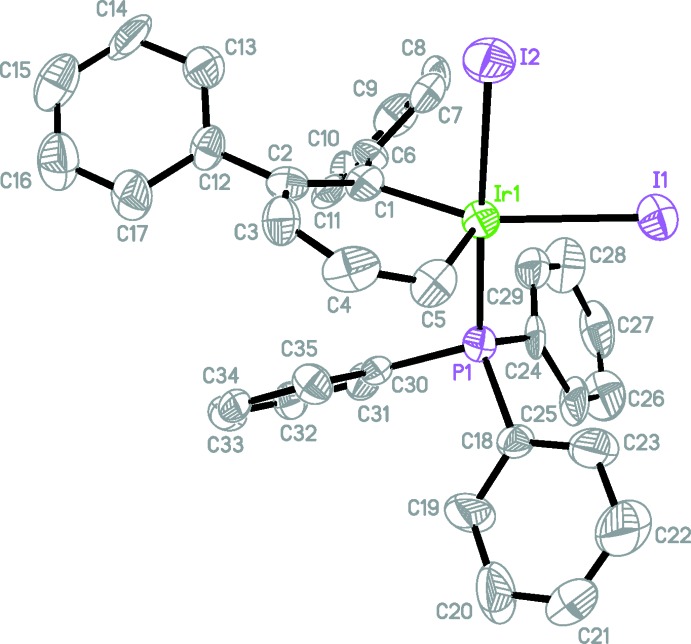
The mol­ecular structure of (II), showing the atom-labelling scheme. Displacement ellipsoids are drawn at the 50% probability level.

Compounds (I), [IrBr_2_(C_17_H_13_)(C_18_H_15_P)], and (II), [IrI_2_(C_17_H_13_)(C_18_H_15_P)], are isotypic. The mol­ecular structures of (I) (Fig. 1 ▸) and (II) (Fig. 2 ▸) confirm that Ir^III^ is five-coordinated in these complexes with only one tri­phenyl­phosphine group bound to the iridium atom, unambiguously proving that the mol­ecules are indeed 16-electron, high-oxidation-state irida­benzenes. The coordination geometry of the Ir^III^ atom in both structures can be best described as a distorted square pyramid with the P1, Br1(I1), Br2(I2) and C1 atoms in the basal plane and the C5 atom in the apical position. The Br1(I1), Br2(I2), P1, C1 fragments are planar within 0.17 Å (Br) and 0.21 Å (I) and the Ir atom is out on 0.22 Å (Br) and 0.24 Å (I) from the average planes of this fragment. The C—C bond lengths in the benzene rings in both structures range from 1.360 (15) to 1.402 (16) Å [average 1.387 and 1.382 Å in (I) and (II), respectively], indicative of bond homogenization and electron delocalization. Both Ir—C bond lengths [1.958 (5), 1.903 (5) Å, and 1.963 (11), 1.913 (12) Å, respectively, for Ir—C1 and Ir—C5 in (I) and (II)] are shorter than typical Ir^I^ irida­benzenes (2.01–2.05 Å), reflecting the higher Ir^III^ oxidation state (Fernández & Frenking, 2007 ▸).

**Figure 3:**
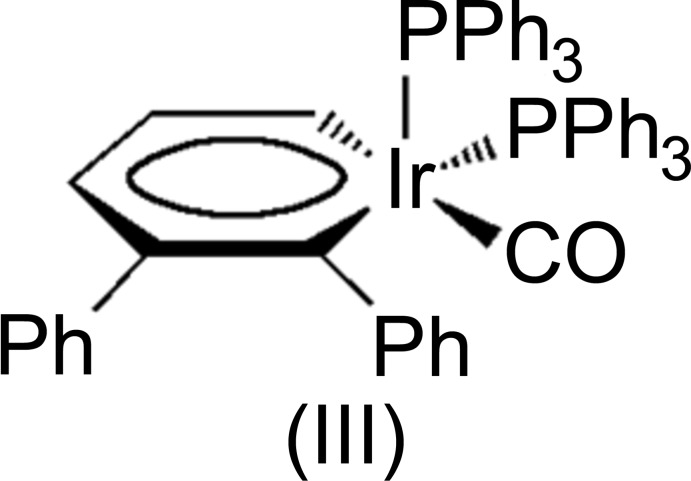
Scheme of irida­benzene (III) employed as an educt

Additionally, the irida­benzene ring in both structures significantly deviates from planarity (Zhu *et al.*, 2007 ▸); the dihedral angle between the C1–C5 fragments [which are planar within 0.03 and 0.04 Å, respectively, in (I) and (II)] and the C1—Ir1—C5 plane is 17.2 (3)° in (I) and 14.9 (7)° in (II). In both structures the open coordination site is located equatorially to the iridium atom, as manifested by the extremely large Br1(I1)—Ir—C1 bond angle of 158.5 (2) [156.0 (3)]° {*cf*, Br1(I1)—Ir—C5, 110.5 (2) [113.6 (4)°]}. This site is typically occupied by CO in all of our previous irida­benzene studies, such as (III) (Fig. 3 ▸). The steric bulk of the two halogen atoms, the tri­phenyl­phosphine group, and the phenyl moiety located on C1 all contribute to the presence of the apparent open coordination site. We did consider the possibility of an H atom or H_2_ mol­ecule occupying the open coordination site. The distance Ir1⋯H29*A* (one of the H atoms on the closest phenyl group) in (I) is *ca* 3.18 Å. This H29*A* atom is on the opposite side from the C5 atom (the C5—Ir1⋯H29*A* angle is 147°). If present, the Ir—H distance would be around 1.5–1.6 Å. In such a case, the distance between this H atom and the H29*A* atom from the phenyl ring should be 1.6–1.7 Å. This distance is too short as a typical H⋯H contact is 2.4 Å. It follows then that if one H atom does not fit, H_2_ will not either. The displacement parameters of most C atoms in the phenyl rings are elongated perpendicular to the average plane of the Ph rings showing their flexibility or statistical disorder.

## Supra­molecular features   

**Figure 4:**
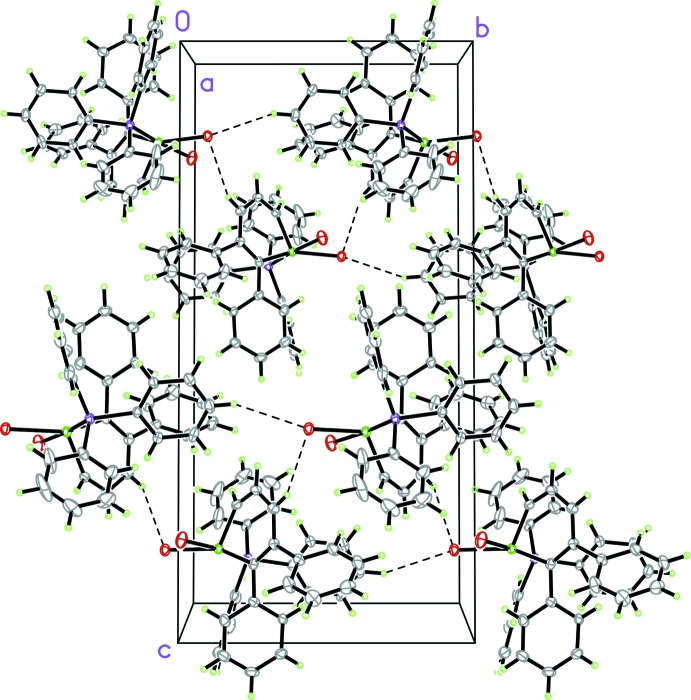
A fragment of the crystal structure of (I) in a view along [100], showing association of the mol­ecules in the crystal packing by weak C—H⋯Br inter­actions (dashed lines). Atom labels are omitted for clarity. The crystal of (II) is isostructural with the crystal of (I).

Compounds (I) and (II) are typical mol­ecular crystals without specific supra­molecular features. Additionally to van der Waals forces, in these structures there are some weak C—H⋯*X* (*X* = Br, I) inter­actions with C⋯*X* distances in the ranges of 3.533 (7)– 3.717 (5) and 3.699 (17)–3.707 (12) Å, respectively, for Br and I (Tables 1 ▸ and 2 ▸). A fragment of the crystal structure of (I) is given in Fig. 4 ▸, illustrating one such weak inter­action.

## Synthesis and crystallization   

Reaction of irida­benzene (Gilbertson *et al.*, 1999 ▸), (III) (Fig. 3 ▸) with one equivalent of bromine at 195 K produced a dark-brown solution that was warmed to 273 K over a period of 30 min. Recrystallization from acetone at 243 K afforded bluish brown crystals of (I). Similarly, reaction of (III) with iodine at 195 K also produced a dark-brown solution containing (II) which was crystallized in similar conditions to give bluish brown crystals. While (I) and (II) were stable in the solid state for weeks at 243 K without noticeable decomposition, solutions of either of the irida­benzenes degraded rapidly and thus made their complete characterization extremely challenging.

## Refinement   

Crystal data, data collection and structure refinement details are summarized in Table 3 ▸. All H atoms were positioned geometrically and refined in a rigid-group model with C—H = 0.95 Å, *U*_iso_(H) = 1.2*U*_eq_(C).

## Supplementary Material

Crystal structure: contains datablock(s) I, II. DOI: 10.1107/S2056989015018952/wm5215sup1.cif

Structure factors: contains datablock(s) I. DOI: 10.1107/S2056989015018952/wm5215Isup2.hkl

Structure factors: contains datablock(s) II. DOI: 10.1107/S2056989015018952/wm5215IIsup3.hkl

CCDC references: 1430096, 1430095

Additional supporting information:  crystallographic information; 3D view; checkCIF report

## Figures and Tables

**Table 1:** Hydrogen-bond geometry (Å, °) for (I)

*D*—H⋯*A*	*D*—H	H⋯*A*	*D*⋯*A*	*D*—H⋯*A*
C3—H3*A*⋯Br1^i^	0.95	2.87	3.717 (5)	149
C7—H7*A*⋯Br2	0.95	2.85	3.609 (7)	137
C23—H23*A*⋯Br1	0.95	2.79	3.533 (7)	135

Symmetry code: (i) 
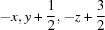
.

**Table 2:** Hydrogen-bond geometry (Å, °) for (II)

*D*—H⋯*A*	*D*—H	H⋯*A*	*D*⋯*A*	*D*—H⋯*A*
C7—H7*A*⋯I2	0.95	2.94	3.707 (12)	138
C23—H23*A*⋯I1	0.95	2.84	3.699 (17)	152

**Table 3:** Experimental details

	(I)	(II)
Crystal data		
Chemical formula	[IrBr_2_(C_17_H_13_)(C_18_H_15_P)]	[Ir(C_17_H_13_)I_2_(C_18_H_15_P)]
*M*_r_	831.56	925.54
Crystal system, space group	Monoclinic, *P*2_1_/*c*	Monoclinic, *P*2_1_/*c*
Temperature (K)	173	173
*a*, *b*, *c* (Å)	10.6200 (8), 11.6901 (8), 23.8782 (17)	10.5973 (14), 11.9431 (16), 24.457 (3)
β (°)	95.094 (2)	93.331 (3)
*V* (Å^3^)	2952.7 (4)	3090.1 (7)
*Z*	4	4
Radiation type	Mo *K*α	Mo *K*α
μ (mm^−1^)	7.31	6.39
Crystal size (mm)	0.09 × 0.07 × 0.04	0.07 × 0.06 × 0.05

Data collection		
Diffractometer	Bruker *APEX* CCD	Bruker *APEX* CCD
Absorption correction	Multi-scan (*SADABS*; Bruker, 2008 ▸)	Multi-scan (*SADABS*; Bruker, 2008 ▸)
*T*_min_, *T*_max_	0.822, 1.000	0.844, 1.000
No. of measured, independent and observed [*I* > 2σ(*I*)] reflections	32559, 6439, 5040	29241, 5435, 3440
*R*_int_	0.063	0.132
(sin θ/λ)_max_ (Å^−1^)	0.639	0.595

Refinement		
*R*[*F* ^2^ > 2σ(*F* ^2^)], *wR*(*F* ^2^), *S*	0.037, 0.079, 1.02	0.056, 0.115, 1.00
No. of reflections	6439	5435
No. of parameters	352	352
H-atom treatment	H-atom parameters constrained	H-atom parameters constrained
Δρ_max_, Δρ_min_ (e Å^−3^)	1.06, −0.65	1.16, −1.15

Computer programs: *APEX2* and *SAINT* (Bruker, 2008 ▸), *SHELXTL* (Sheldrick, 2008 ▸) and *SHELXL2013* (Sheldrick, 2015 ▸).

## supplementary crystallographic information

### (I) Dibromido(1,2-diphenylpenta-1,3-dien-1-yl-5-ylidene)(triphenylphosphane)iridium(III) Crystal data

**Table:**

[IrBr_2_(C_17_H_13_)(C_18_H_15_P)]	*F*(000) = 1600
*M**_r_* = 831.56	*D*_x_ = 1.871 Mg m^−^^3^
Monoclinic, *P*2_1_/*c*	Mo *K*α radiation, λ = 0.71073 Å
*a* = 10.6200 (8) Å	Cell parameters from 3916 reflections
*b* = 11.6901 (8) Å	θ = 2.4–22.0°
*c* = 23.8782 (17) Å	µ = 7.31 mm^−^^1^
β = 95.094 (2)°	*T* = 173 K
*V* = 2952.7 (4) Å^3^	Cut-block, blue
*Z* = 4	0.09 × 0.07 × 0.04 mm

### (I) Dibromido(1,2-diphenylpenta-1,3-dien-1-yl-5-ylidene)(triphenylphosphane)iridium(III) Data collection

**Table:**

Bruker APEX CCD diffractometer	5040 reflections with *I* > 2σ(*I*)
Radiation source: sealed tube	*R*_int_ = 0.063
phi and ω scans	θ_max_ = 27.0°, θ_min_ = 1.7°
Absorption correction: multi-scan (*SADABS*; Bruker, 2008)	*h* = −13→13
*T*_min_ = 0.822, *T*_max_ = 1.000	*k* = −14→14
32559 measured reflections	*l* = −30→30
6439 independent reflections	

### (I) Dibromido(1,2-diphenylpenta-1,3-dien-1-yl-5-ylidene)(triphenylphosphane)iridium(III) Refinement

**Table:**

Refinement on *F*^2^	0 restraints
Least-squares matrix: full	Hydrogen site location: inferred from neighbouring sites
*R*[*F*^2^ > 2σ(*F*^2^)] = 0.037	H-atom parameters constrained
*wR*(*F*^2^) = 0.079	*w* = 1/[σ^2^(*F*_o_^2^) + (0.0341*P*)^2^] where *P* = (*F*_o_^2^ + 2*F*_c_^2^)/3
*S* = 1.02	(Δ/σ)_max_ = 0.003
6439 reflections	Δρ_max_ = 1.06 e Å^−^^3^
352 parameters	Δρ_min_ = −0.65 e Å^−^^3^

### (I) Dibromido(1,2-diphenylpenta-1,3-dien-1-yl-5-ylidene)(triphenylphosphane)iridium(III) Special details

**Table:**

Geometry. All e.s.d.'s (except the e.s.d. in the dihedral angle between two l.s. planes) are estimated using the full covariance matrix. The cell e.s.d.'s are taken into account individually in the estimation of e.s.d.'s in distances, angles and torsion angles; correlations between e.s.d.'s in cell parameters are only used when they are defined by crystal symmetry. An approximate (isotropic) treatment of cell e.s.d.'s is used for estimating e.s.d.'s involving l.s. planes.

### (I) Dibromido(1,2-diphenylpenta-1,3-dien-1-yl-5-ylidene)(triphenylphosphane)iridium(III) Fractional atomic coordinates and isotropic or equivalent isotropic displacement parameters (Å^2^)

**Table:**

	*x*	*y*	*z*	*U*_iso_*/*U*_eq_	
Ir1	0.10176 (2)	0.13179 (2)	0.85083 (2)	0.02518 (7)	
Br1	0.22033 (5)	−0.05824 (5)	0.85942 (2)	0.03303 (14)	
Br2	−0.09573 (6)	0.01646 (5)	0.82570 (3)	0.04562 (17)	
P1	0.28971 (13)	0.22577 (11)	0.87771 (6)	0.0256 (3)	
C1	−0.0134 (5)	0.2540 (4)	0.8696 (2)	0.0263 (12)	
C2	−0.0869 (5)	0.3252 (5)	0.8329 (2)	0.0286 (12)	
C3	−0.0730 (5)	0.3229 (5)	0.7752 (2)	0.0342 (13)	
H3A	−0.1305	0.3675	0.7516	0.041*	
C4	0.0175 (5)	0.2611 (5)	0.7493 (2)	0.0355 (14)	
H4A	0.0171	0.2663	0.7096	0.043*	
C5	0.1068 (5)	0.1935 (4)	0.7773 (2)	0.0299 (13)	
H5A	0.1789	0.1754	0.7582	0.036*	
C6	−0.0148 (5)	0.2567 (5)	0.9317 (2)	0.0289 (12)	
C7	−0.0513 (5)	0.1597 (5)	0.9599 (3)	0.0378 (14)	
H7A	−0.0838	0.0953	0.9392	0.045*	
C8	−0.0404 (6)	0.1568 (6)	1.0184 (3)	0.0461 (17)	
H8A	−0.0658	0.0907	1.0376	0.055*	
C9	0.0069 (6)	0.2492 (6)	1.0481 (3)	0.0461 (17)	
H9A	0.0157	0.2463	1.0880	0.055*	
C10	0.0424 (6)	0.3469 (5)	1.0209 (2)	0.0400 (15)	
H10A	0.0762	0.4102	1.0422	0.048*	
C11	0.0287 (5)	0.3527 (4)	0.9628 (2)	0.0282 (12)	
H11A	0.0487	0.4212	0.9442	0.034*	
C12	−0.1768 (5)	0.4095 (5)	0.8531 (2)	0.0306 (13)	
C13	−0.2696 (6)	0.3785 (5)	0.8876 (3)	0.0470 (17)	
H13A	−0.2729	0.3018	0.9006	0.056*	
C14	−0.3575 (6)	0.4567 (6)	0.9036 (3)	0.0538 (19)	
H14A	−0.4215	0.4331	0.9265	0.065*	
C15	−0.3523 (6)	0.5686 (6)	0.8863 (3)	0.0522 (18)	
H15A	−0.4129	0.6223	0.8969	0.063*	
C16	−0.2586 (7)	0.6027 (6)	0.8534 (3)	0.0549 (19)	
H16A	−0.2535	0.6803	0.8421	0.066*	
C17	−0.1716 (6)	0.5231 (5)	0.8368 (3)	0.0436 (16)	
H17A	−0.1077	0.5471	0.8140	0.052*	
C18	0.4083 (5)	0.2172 (5)	0.8265 (2)	0.0317 (13)	
C19	0.4459 (7)	0.3103 (6)	0.7980 (3)	0.060 (2)	
H19A	0.4096	0.3828	0.8047	0.072*	
C20	0.5357 (7)	0.3019 (8)	0.7595 (3)	0.079 (3)	
H20A	0.5600	0.3680	0.7400	0.094*	
C21	0.5881 (7)	0.2004 (7)	0.7499 (3)	0.070 (2)	
H21A	0.6472	0.1937	0.7225	0.085*	
C22	0.5567 (8)	0.1074 (7)	0.7793 (5)	0.109 (4)	
H22A	0.5966	0.0362	0.7737	0.131*	
C23	0.4670 (8)	0.1157 (6)	0.8173 (4)	0.093 (3)	
H23A	0.4456	0.0497	0.8376	0.111*	
C24	0.3671 (5)	0.1769 (4)	0.9445 (2)	0.0315 (13)	
C25	0.4976 (6)	0.1792 (5)	0.9555 (3)	0.0409 (15)	
H25A	0.5494	0.1978	0.9263	0.049*	
C26	0.5528 (6)	0.1543 (5)	1.0092 (3)	0.0518 (19)	
H26A	0.6421	0.1561	1.0169	0.062*	
C27	0.4770 (8)	0.1269 (5)	1.0513 (3)	0.057 (2)	
H27A	0.5147	0.1097	1.0879	0.068*	
C28	0.3482 (7)	0.1243 (5)	1.0410 (3)	0.0508 (18)	
H28A	0.2969	0.1064	1.0704	0.061*	
C29	0.2929 (6)	0.1479 (5)	0.9874 (2)	0.0363 (14)	
H29A	0.2036	0.1442	0.9801	0.044*	
C30	0.2706 (5)	0.3799 (4)	0.8877 (2)	0.0246 (11)	
C31	0.3186 (5)	0.4357 (5)	0.9360 (2)	0.0311 (13)	
H31A	0.3602	0.3934	0.9662	0.037*	
C32	0.3061 (5)	0.5530 (5)	0.9405 (3)	0.0389 (15)	
H32A	0.3389	0.5908	0.9738	0.047*	
C33	0.2464 (6)	0.6157 (5)	0.8970 (3)	0.0410 (15)	
H33A	0.2399	0.6964	0.9002	0.049*	
C34	0.1966 (5)	0.5618 (5)	0.8493 (3)	0.0369 (15)	
H34A	0.1540	0.6047	0.8196	0.044*	
C35	0.2082 (5)	0.4438 (4)	0.8444 (2)	0.0290 (12)	
H35A	0.1734	0.4065	0.8113	0.035*	

### (I) Dibromido(1,2-diphenylpenta-1,3-dien-1-yl-5-ylidene)(triphenylphosphane)iridium(III) Atomic displacement parameters (Å^2^)

**Table:**

	*U*^11^	*U*^22^	*U*^33^	*U*^12^	*U*^13^	*U*^23^
Ir1	0.02630 (12)	0.02190 (11)	0.02676 (12)	0.00155 (9)	−0.00084 (8)	−0.00056 (9)
Br1	0.0366 (3)	0.0245 (3)	0.0364 (3)	0.0044 (2)	−0.0057 (3)	0.0009 (2)
Br2	0.0350 (4)	0.0346 (3)	0.0655 (5)	−0.0047 (3)	−0.0057 (3)	−0.0103 (3)
P1	0.0256 (8)	0.0216 (7)	0.0292 (8)	0.0029 (6)	0.0012 (6)	−0.0005 (6)
C1	0.022 (3)	0.024 (3)	0.032 (3)	−0.002 (2)	0.003 (2)	−0.002 (2)
C2	0.020 (3)	0.030 (3)	0.035 (3)	0.002 (2)	−0.001 (2)	0.002 (3)
C3	0.034 (3)	0.037 (3)	0.031 (3)	0.002 (3)	−0.001 (3)	0.004 (3)
C4	0.045 (4)	0.035 (3)	0.025 (3)	−0.006 (3)	−0.002 (3)	0.001 (3)
C5	0.034 (3)	0.030 (3)	0.026 (3)	0.003 (2)	0.003 (2)	−0.005 (2)
C6	0.025 (3)	0.033 (3)	0.030 (3)	0.011 (2)	0.008 (2)	0.007 (3)
C7	0.037 (3)	0.031 (3)	0.046 (4)	0.010 (3)	0.010 (3)	0.006 (3)
C8	0.057 (4)	0.046 (4)	0.038 (4)	0.017 (3)	0.017 (3)	0.015 (3)
C9	0.059 (4)	0.053 (4)	0.028 (3)	0.023 (4)	0.015 (3)	0.007 (3)
C10	0.050 (4)	0.040 (4)	0.032 (3)	0.014 (3)	0.010 (3)	−0.002 (3)
C11	0.030 (3)	0.024 (3)	0.031 (3)	0.010 (2)	0.006 (2)	0.003 (2)
C12	0.022 (3)	0.035 (3)	0.033 (3)	0.007 (2)	−0.002 (2)	0.006 (3)
C13	0.030 (3)	0.038 (4)	0.074 (5)	0.004 (3)	0.010 (3)	0.013 (3)
C14	0.032 (4)	0.062 (5)	0.070 (5)	0.015 (3)	0.017 (3)	0.009 (4)
C15	0.045 (4)	0.063 (5)	0.049 (4)	0.026 (4)	0.004 (3)	−0.003 (4)
C16	0.074 (5)	0.038 (4)	0.054 (4)	0.022 (4)	0.012 (4)	0.013 (3)
C17	0.049 (4)	0.040 (4)	0.044 (4)	0.013 (3)	0.014 (3)	0.013 (3)
C18	0.024 (3)	0.031 (3)	0.040 (3)	0.002 (2)	0.006 (3)	−0.012 (3)
C19	0.076 (5)	0.049 (4)	0.060 (5)	0.021 (4)	0.039 (4)	0.017 (4)
C20	0.081 (6)	0.092 (7)	0.071 (6)	0.035 (5)	0.048 (5)	0.028 (5)
C21	0.053 (5)	0.084 (6)	0.080 (6)	−0.006 (5)	0.041 (4)	−0.035 (5)
C22	0.088 (7)	0.057 (6)	0.198 (11)	−0.014 (5)	0.099 (8)	−0.061 (6)
C23	0.089 (6)	0.033 (4)	0.169 (10)	−0.012 (4)	0.090 (7)	−0.022 (5)
C24	0.035 (3)	0.020 (3)	0.036 (3)	0.007 (2)	−0.012 (3)	−0.006 (2)
C25	0.038 (4)	0.024 (3)	0.058 (4)	−0.001 (3)	−0.012 (3)	0.001 (3)
C26	0.043 (4)	0.033 (4)	0.072 (5)	0.005 (3)	−0.034 (4)	−0.011 (3)
C27	0.079 (6)	0.036 (4)	0.048 (4)	0.020 (4)	−0.037 (4)	−0.008 (3)
C28	0.070 (5)	0.045 (4)	0.036 (4)	0.024 (4)	−0.006 (3)	−0.002 (3)
C29	0.037 (3)	0.040 (4)	0.030 (3)	0.012 (3)	−0.005 (3)	−0.003 (3)
C30	0.024 (3)	0.021 (3)	0.030 (3)	0.002 (2)	0.007 (2)	0.000 (2)
C31	0.036 (3)	0.025 (3)	0.032 (3)	0.004 (2)	0.001 (3)	−0.002 (2)
C32	0.043 (4)	0.031 (3)	0.043 (4)	−0.004 (3)	0.007 (3)	−0.012 (3)
C33	0.045 (4)	0.027 (3)	0.054 (4)	0.001 (3)	0.020 (3)	−0.004 (3)
C34	0.029 (3)	0.025 (3)	0.059 (4)	0.003 (3)	0.012 (3)	0.013 (3)
C35	0.027 (3)	0.025 (3)	0.034 (3)	0.000 (2)	0.001 (2)	0.003 (2)

### (I) Dibromido(1,2-diphenylpenta-1,3-dien-1-yl-5-ylidene)(triphenylphosphane)iridium(III) Geometric parameters (Å, º)

**Table:**

Ir1—C5	1.903 (5)	C16—C17	1.393 (8)
Ir1—C1	1.958 (5)	C16—H16A	0.9500
Ir1—P1	2.3185 (14)	C17—H17A	0.9500
Ir1—Br2	2.5205 (6)	C18—C19	1.362 (8)
Ir1—Br1	2.5528 (6)	C18—C23	1.367 (8)
P1—C24	1.819 (5)	C19—C20	1.387 (9)
P1—C30	1.831 (5)	C19—H19A	0.9500
P1—C18	1.833 (5)	C20—C21	1.339 (10)
C1—C2	1.396 (7)	C20—H20A	0.9500
C1—C6	1.486 (7)	C21—C22	1.351 (12)
C2—C3	1.399 (7)	C21—H21A	0.9500
C2—C12	1.482 (7)	C22—C23	1.376 (10)
C3—C4	1.389 (8)	C22—H22A	0.9500
C3—H3A	0.9500	C23—H23A	0.9500
C4—C5	1.363 (7)	C24—C25	1.388 (7)
C4—H4A	0.9500	C24—C29	1.390 (8)
C5—H5A	0.9500	C25—C26	1.394 (8)
C6—C7	1.391 (7)	C25—H25A	0.9500
C6—C11	1.402 (7)	C26—C27	1.379 (10)
C7—C8	1.391 (8)	C26—H26A	0.9500
C7—H7A	0.9500	C27—C28	1.368 (9)
C8—C9	1.363 (9)	C27—H27A	0.9500
C8—H8A	0.9500	C28—C29	1.387 (8)
C9—C10	1.382 (8)	C28—H28A	0.9500
C9—H9A	0.9500	C29—H29A	0.9500
C10—C11	1.384 (7)	C30—C31	1.383 (7)
C10—H10A	0.9500	C30—C35	1.395 (7)
C11—H11A	0.9500	C31—C32	1.383 (7)
C12—C17	1.387 (8)	C31—H31A	0.9500
C12—C13	1.388 (8)	C32—C33	1.378 (8)
C13—C14	1.384 (8)	C32—H32A	0.9500
C13—H13A	0.9500	C33—C34	1.366 (8)
C14—C15	1.374 (9)	C33—H33A	0.9500
C14—H14A	0.9500	C34—C35	1.390 (7)
C15—C16	1.379 (9)	C34—H34A	0.9500
C15—H15A	0.9500	C35—H35A	0.9500
			
C5—Ir1—C1	90.2 (2)	C15—C16—C17	119.9 (6)
C5—Ir1—P1	88.94 (17)	C15—C16—H16A	120.0
C1—Ir1—P1	97.51 (15)	C17—C16—H16A	120.0
C5—Ir1—Br2	94.26 (16)	C12—C17—C16	121.0 (6)
C1—Ir1—Br2	85.49 (15)	C12—C17—H17A	119.5
P1—Ir1—Br2	175.61 (4)	C16—C17—H17A	119.5
C5—Ir1—Br1	110.50 (16)	C19—C18—C23	117.1 (6)
C1—Ir1—Br1	158.47 (15)	C19—C18—P1	122.5 (4)
P1—Ir1—Br1	89.07 (4)	C23—C18—P1	120.3 (5)
Br2—Ir1—Br1	87.01 (2)	C18—C19—C20	121.6 (7)
C24—P1—C30	104.0 (2)	C18—C19—H19A	119.2
C24—P1—C18	106.4 (3)	C20—C19—H19A	119.2
C30—P1—C18	103.4 (2)	C21—C20—C19	119.8 (8)
C24—P1—Ir1	113.86 (19)	C21—C20—H20A	120.1
C30—P1—Ir1	113.56 (17)	C19—C20—H20A	120.1
C18—P1—Ir1	114.52 (18)	C20—C21—C22	119.9 (7)
C2—C1—C6	124.0 (5)	C20—C21—H21A	120.1
C2—C1—Ir1	128.2 (4)	C22—C21—H21A	120.1
C6—C1—Ir1	107.8 (3)	C21—C22—C23	120.2 (7)
C1—C2—C3	120.3 (5)	C21—C22—H22A	119.9
C1—C2—C12	122.2 (5)	C23—C22—H22A	119.9
C3—C2—C12	117.5 (5)	C18—C23—C22	121.3 (8)
C4—C3—C2	125.8 (5)	C18—C23—H23A	119.3
C4—C3—H3A	117.1	C22—C23—H23A	119.3
C2—C3—H3A	117.1	C25—C24—C29	119.1 (5)
C5—C4—C3	124.2 (5)	C25—C24—P1	121.7 (5)
C5—C4—H4A	117.9	C29—C24—P1	118.8 (4)
C3—C4—H4A	117.9	C24—C25—C26	120.1 (6)
C4—C5—Ir1	126.6 (4)	C24—C25—H25A	119.9
C4—C5—H5A	116.7	C26—C25—H25A	119.9
Ir1—C5—H5A	116.7	C27—C26—C25	119.6 (6)
C7—C6—C11	119.2 (5)	C27—C26—H26A	120.2
C7—C6—C1	119.6 (5)	C25—C26—H26A	120.2
C11—C6—C1	121.0 (5)	C28—C27—C26	120.9 (6)
C8—C7—C6	120.3 (6)	C28—C27—H27A	119.6
C8—C7—H7A	119.9	C26—C27—H27A	119.6
C6—C7—H7A	119.9	C27—C28—C29	119.7 (7)
C9—C8—C7	119.8 (6)	C27—C28—H28A	120.2
C9—C8—H8A	120.1	C29—C28—H28A	120.2
C7—C8—H8A	120.1	C28—C29—C24	120.5 (6)
C8—C9—C10	121.0 (6)	C28—C29—H29A	119.7
C8—C9—H9A	119.5	C24—C29—H29A	119.7
C10—C9—H9A	119.5	C31—C30—C35	118.8 (5)
C9—C10—C11	120.1 (6)	C31—C30—P1	122.4 (4)
C9—C10—H10A	120.0	C35—C30—P1	118.8 (4)
C11—C10—H10A	120.0	C32—C31—C30	120.1 (5)
C10—C11—C6	119.6 (5)	C32—C31—H31A	120.0
C10—C11—H11A	120.2	C30—C31—H31A	120.0
C6—C11—H11A	120.2	C33—C32—C31	120.7 (6)
C17—C12—C13	117.7 (5)	C33—C32—H32A	119.6
C17—C12—C2	120.2 (5)	C31—C32—H32A	119.6
C13—C12—C2	122.1 (5)	C34—C33—C32	120.0 (6)
C14—C13—C12	121.5 (6)	C34—C33—H33A	120.0
C14—C13—H13A	119.2	C32—C33—H33A	120.0
C12—C13—H13A	119.2	C33—C34—C35	119.8 (6)
C15—C14—C13	119.9 (6)	C33—C34—H34A	120.1
C15—C14—H14A	120.0	C35—C34—H34A	120.1
C13—C14—H14A	120.0	C34—C35—C30	120.6 (5)
C14—C15—C16	119.8 (6)	C34—C35—H35A	119.7
C14—C15—H15A	120.1	C30—C35—H35A	119.7
C16—C15—H15A	120.1		
			
C6—C1—C2—C3	175.3 (5)	Ir1—P1—C18—C23	−69.8 (7)
Ir1—C1—C2—C3	−7.5 (8)	C23—C18—C19—C20	2.8 (12)
C6—C1—C2—C12	−1.5 (8)	P1—C18—C19—C20	−180.0 (6)
Ir1—C1—C2—C12	175.8 (4)	C18—C19—C20—C21	−0.4 (13)
C1—C2—C3—C4	−5.7 (9)	C19—C20—C21—C22	−2.5 (14)
C12—C2—C3—C4	171.1 (5)	C20—C21—C22—C23	2.8 (16)
C2—C3—C4—C5	−0.3 (10)	C19—C18—C23—C22	−2.4 (13)
C3—C4—C5—Ir1	19.7 (9)	P1—C18—C23—C22	−179.7 (8)
C2—C1—C6—C7	119.0 (6)	C21—C22—C23—C18	−0.3 (17)
Ir1—C1—C6—C7	−58.8 (6)	C30—P1—C24—C25	−86.5 (5)
C2—C1—C6—C11	−65.7 (7)	C18—P1—C24—C25	22.3 (5)
Ir1—C1—C6—C11	116.5 (4)	Ir1—P1—C24—C25	149.4 (4)
C11—C6—C7—C8	−2.2 (8)	C30—P1—C24—C29	86.7 (5)
C1—C6—C7—C8	173.1 (5)	C18—P1—C24—C29	−164.5 (4)
C6—C7—C8—C9	−0.4 (9)	Ir1—P1—C24—C29	−37.4 (5)
C7—C8—C9—C10	1.1 (10)	C29—C24—C25—C26	−0.9 (8)
C8—C9—C10—C11	0.8 (9)	P1—C24—C25—C26	172.3 (4)
C9—C10—C11—C6	−3.4 (8)	C24—C25—C26—C27	0.2 (9)
C7—C6—C11—C10	4.1 (8)	C25—C26—C27—C28	−0.2 (10)
C1—C6—C11—C10	−171.2 (5)	C26—C27—C28—C29	0.9 (10)
C1—C2—C12—C17	127.5 (6)	C27—C28—C29—C24	−1.6 (9)
C3—C2—C12—C17	−49.3 (8)	C25—C24—C29—C28	1.6 (8)
C1—C2—C12—C13	−53.7 (8)	P1—C24—C29—C28	−171.8 (4)
C3—C2—C12—C13	129.5 (6)	C24—P1—C30—C31	4.5 (5)
C17—C12—C13—C14	2.5 (10)	C18—P1—C30—C31	−106.5 (5)
C2—C12—C13—C14	−176.3 (6)	Ir1—P1—C30—C31	128.8 (4)
C12—C13—C14—C15	−1.5 (11)	C24—P1—C30—C35	−177.2 (4)
C13—C14—C15—C16	−0.5 (11)	C18—P1—C30—C35	71.8 (5)
C14—C15—C16—C17	1.4 (11)	Ir1—P1—C30—C35	−52.9 (4)
C13—C12—C17—C16	−1.6 (10)	C35—C30—C31—C32	−1.0 (8)
C2—C12—C17—C16	177.2 (6)	P1—C30—C31—C32	177.3 (4)
C15—C16—C17—C12	−0.4 (11)	C30—C31—C32—C33	−0.3 (9)
C24—P1—C18—C19	−120.2 (6)	C31—C32—C33—C34	1.4 (9)
C30—P1—C18—C19	−11.0 (6)	C32—C33—C34—C35	−1.2 (8)
Ir1—P1—C18—C19	113.1 (5)	C33—C34—C35—C30	−0.1 (8)
C24—P1—C18—C23	56.9 (7)	C31—C30—C35—C34	1.2 (8)
C30—P1—C18—C23	166.1 (6)	P1—C30—C35—C34	−177.2 (4)

### (I) Dibromido(1,2-diphenylpenta-1,3-dien-1-yl-5-ylidene)(triphenylphosphane)iridium(III) Hydrogen-bond geometry (Å, º)

**Table:**

*D*—H···*A*	*D*—H	H···*A*	*D*···*A*	*D*—H···*A*
C3—H3*A*···Br1^i^	0.95	2.87	3.717 (5)	149
C7—H7*A*···Br2	0.95	2.85	3.609 (7)	137
C23—H23*A*···Br1	0.95	2.79	3.533 (7)	135

Symmetry code: (i) −*x*, *y*+1/2, −*z*+3/2.

### (II) (1,2-Diphenylpenta-1,3-dien-1-yl-5-ylidene)diiodido(triphenylphosphane)iridium(III) Crystal data

**Table:**

[Ir(C_17_H_13_)I_2_(C_18_H_15_P)]	*F*(000) = 1744
*M**_r_* = 925.54	*D*_x_ = 1.989 Mg m^−^^3^
Monoclinic, *P*2_1_/*c*	Mo *K*α radiation, λ = 0.71073 Å
*a* = 10.5973 (14) Å	Cell parameters from 1109 reflections
*b* = 11.9431 (16) Å	θ = 2.4–15.6°
*c* = 24.457 (3) Å	µ = 6.39 mm^−^^1^
β = 93.331 (3)°	*T* = 173 K
*V* = 3090.1 (7) Å^3^	Cut-block, dark-blue
*Z* = 4	0.07 × 0.06 × 0.05 mm

### (II) (1,2-Diphenylpenta-1,3-dien-1-yl-5-ylidene)diiodido(triphenylphosphane)iridium(III) Data collection

**Table:**

Bruker APEX CCD diffractometer	3440 reflections with *I* > 2σ(*I*)
Radiation source: sealed tube	*R*_int_ = 0.132
phi and ω scans	θ_max_ = 25.0°, θ_min_ = 1.7°
Absorption correction: multi-scan (*SADABS*; Bruker, 2008)	*h* = −12→12
*T*_min_ = 0.844, *T*_max_ = 1.000	*k* = −14→14
29241 measured reflections	*l* = −29→29
5435 independent reflections	

### (II) (1,2-Diphenylpenta-1,3-dien-1-yl-5-ylidene)diiodido(triphenylphosphane)iridium(III) Refinement

**Table:**

Refinement on *F*^2^	0 restraints
Least-squares matrix: full	Hydrogen site location: inferred from neighbouring sites
*R*[*F*^2^ > 2σ(*F*^2^)] = 0.056	H-atom parameters constrained
*wR*(*F*^2^) = 0.115	*w* = 1/[σ^2^(*F*_o_^2^) + (0.0386*P*)^2^] where *P* = (*F*_o_^2^ + 2*F*_c_^2^)/3
*S* = 1.00	(Δ/σ)_max_ < 0.001
5435 reflections	Δρ_max_ = 1.16 e Å^−^^3^
352 parameters	Δρ_min_ = −1.15 e Å^−^^3^

### (II) (1,2-Diphenylpenta-1,3-dien-1-yl-5-ylidene)diiodido(triphenylphosphane)iridium(III) Special details

**Table:**

Geometry. All e.s.d.'s (except the e.s.d. in the dihedral angle between two l.s. planes) are estimated using the full covariance matrix. The cell e.s.d.'s are taken into account individually in the estimation of e.s.d.'s in distances, angles and torsion angles; correlations between e.s.d.'s in cell parameters are only used when they are defined by crystal symmetry. An approximate (isotropic) treatment of cell e.s.d.'s is used for estimating e.s.d.'s involving l.s. planes.

### (II) (1,2-Diphenylpenta-1,3-dien-1-yl-5-ylidene)diiodido(triphenylphosphane)iridium(III) Fractional atomic coordinates and isotropic or equivalent isotropic displacement parameters (Å^2^)

**Table:**

	*x*	*y*	*z*	*U*_iso_*/*U*_eq_	
Ir1	−0.40123 (5)	−0.36174 (4)	0.34947 (2)	0.03406 (16)	
I1	−0.28307 (8)	−0.56344 (7)	0.36052 (3)	0.0458 (3)	
I2	−0.61957 (8)	−0.47400 (8)	0.32615 (4)	0.0570 (3)	
P1	−0.2104 (3)	−0.2721 (3)	0.37148 (13)	0.0336 (8)	
C11	−0.4688 (10)	−0.1454 (11)	0.4604 (5)	0.041 (3)	
H11A	−0.4502	−0.0779	0.4419	0.050*	
C1	−0.5134 (10)	−0.2398 (10)	0.3700 (5)	0.033 (3)	
C10	−0.4524 (11)	−0.1491 (10)	0.5169 (5)	0.041 (3)	
H10A	−0.4200	−0.0858	0.5366	0.049*	
C30	−0.2297 (10)	−0.1210 (9)	0.3839 (5)	0.029 (3)	
C34	−0.3021 (11)	0.0577 (10)	0.3495 (6)	0.044 (4)	
H34A	−0.3430	0.1017	0.3213	0.053*	
C6	−0.5110 (11)	−0.2361 (10)	0.4302 (5)	0.034 (3)	
C35	−0.2876 (11)	−0.0583 (10)	0.3421 (5)	0.041 (3)	
H35A	−0.3171	−0.0931	0.3088	0.049*	
C18	−0.0961 (10)	−0.2767 (10)	0.3184 (5)	0.033 (3)	
C2	−0.5829 (10)	−0.1661 (10)	0.3362 (5)	0.034 (3)	
C8	−0.5280 (12)	−0.3357 (11)	0.5153 (5)	0.045 (4)	
H8A	−0.5506	−0.4013	0.5345	0.055*	
C7	−0.5417 (10)	−0.3354 (10)	0.4595 (5)	0.040 (3)	
H7A	−0.5713	−0.4003	0.4403	0.048*	
C3	−0.5726 (11)	−0.1673 (10)	0.2803 (5)	0.042 (3)	
H3A	−0.6288	−0.1204	0.2591	0.050*	
C4	−0.4867 (12)	−0.2312 (12)	0.2520 (5)	0.048 (4)	
H4A	−0.4892	−0.2256	0.2132	0.058*	
C12	−0.6710 (11)	−0.0827 (11)	0.3586 (5)	0.037 (3)	
C24	−0.1286 (11)	−0.3182 (9)	0.4345 (5)	0.035 (3)	
C29	−0.1961 (12)	−0.3438 (10)	0.4792 (5)	0.045 (3)	
H29A	−0.2858	−0.3450	0.4751	0.054*	
C32	−0.1985 (11)	0.0448 (10)	0.4383 (6)	0.043 (3)	
H32A	−0.1688	0.0794	0.4716	0.051*	
C33	−0.2572 (12)	0.1074 (11)	0.3972 (6)	0.050 (4)	
H33A	−0.2668	0.1859	0.4019	0.060*	
C9	−0.4831 (12)	−0.2454 (13)	0.5447 (5)	0.055 (4)	
H9A	−0.4731	−0.2485	0.5836	0.066*	
C31	−0.1832 (11)	−0.0687 (10)	0.4310 (5)	0.040 (3)	
H31A	−0.1397	−0.1115	0.4590	0.048*	
C13	−0.7630 (12)	−0.1105 (11)	0.3941 (5)	0.047 (4)	
H13A	−0.7677	−0.1851	0.4074	0.057*	
C5	−0.4000 (11)	−0.3009 (10)	0.2771 (5)	0.043 (3)	
H5A	−0.3308	−0.3219	0.2564	0.051*	
C27	−0.0060 (15)	−0.3660 (11)	0.5353 (7)	0.065 (5)	
H27A	0.0361	−0.3795	0.5700	0.078*	
C17	−0.6678 (13)	0.0281 (11)	0.3407 (5)	0.052 (4)	
H17A	−0.6064	0.0505	0.3161	0.062*	
C25	0.0029 (12)	−0.3220 (10)	0.4405 (6)	0.054 (4)	
H25A	0.0513	−0.3090	0.4096	0.064*	
C28	−0.1381 (15)	−0.3680 (12)	0.5297 (5)	0.061 (4)	
H28A	−0.1867	−0.3857	0.5600	0.073*	
C19	−0.0389 (15)	−0.1850 (13)	0.3015 (7)	0.085 (6)	
H19A	−0.0582	−0.1153	0.3178	0.102*	
C26	0.0632 (14)	−0.3444 (12)	0.4903 (7)	0.068 (5)	
H26A	0.1529	−0.3451	0.4940	0.082*	
C14	−0.8483 (12)	−0.0316 (14)	0.4106 (6)	0.064 (5)	
H14A	−0.9106	−0.0532	0.4349	0.077*	
C21	0.0780 (14)	−0.2803 (14)	0.2371 (7)	0.072 (5)	
H21A	0.1339	−0.2816	0.2081	0.087*	
C15	−0.8452 (14)	0.0770 (14)	0.3927 (6)	0.061 (4)	
H15A	−0.9050	0.1305	0.4038	0.073*	
C16	−0.7538 (15)	0.1058 (13)	0.3586 (6)	0.066 (4)	
H16A	−0.7486	0.1813	0.3466	0.079*	
C20	0.0480 (19)	−0.1864 (14)	0.2609 (7)	0.120 (9)	
H20A	0.0862	−0.1183	0.2504	0.144*	
C23	−0.0627 (17)	−0.3737 (13)	0.2939 (8)	0.118 (8)	
H23A	−0.1018	−0.4418	0.3037	0.142*	
C22	0.0258 (17)	−0.3751 (14)	0.2555 (9)	0.123 (9)	
H22A	0.0515	−0.4451	0.2414	0.148*	

### (II) (1,2-Diphenylpenta-1,3-dien-1-yl-5-ylidene)diiodido(triphenylphosphane)iridium(III) Atomic displacement parameters (Å^2^)

**Table:**

	*U*^11^	*U*^22^	*U*^33^	*U*^12^	*U*^13^	*U*^23^
Ir1	0.0342 (3)	0.0343 (3)	0.0334 (3)	−0.0003 (3)	−0.0009 (2)	0.0000 (3)
I1	0.0508 (6)	0.0394 (5)	0.0467 (6)	0.0009 (4)	−0.0024 (4)	0.0016 (4)
I2	0.0433 (6)	0.0551 (6)	0.0717 (7)	−0.0108 (5)	−0.0057 (5)	−0.0114 (5)
P1	0.0322 (19)	0.0320 (19)	0.0368 (19)	0.0022 (15)	0.0017 (15)	0.0011 (16)
C11	0.028 (7)	0.057 (9)	0.040 (8)	0.013 (7)	0.015 (6)	0.008 (7)
C1	0.023 (6)	0.041 (8)	0.031 (7)	−0.003 (6)	−0.013 (5)	0.001 (6)
C10	0.053 (8)	0.034 (8)	0.036 (8)	0.017 (7)	0.008 (6)	0.005 (7)
C30	0.020 (6)	0.030 (7)	0.038 (7)	0.001 (5)	0.003 (5)	0.004 (6)
C34	0.041 (8)	0.038 (8)	0.056 (9)	0.014 (7)	0.028 (7)	0.013 (7)
C6	0.036 (7)	0.034 (8)	0.034 (7)	−0.003 (6)	0.008 (6)	0.014 (6)
C35	0.046 (8)	0.041 (8)	0.036 (8)	−0.003 (7)	−0.002 (6)	0.003 (6)
C18	0.027 (7)	0.033 (7)	0.041 (8)	−0.004 (6)	0.004 (6)	−0.006 (6)
C2	0.030 (7)	0.037 (8)	0.033 (7)	−0.010 (6)	−0.003 (6)	−0.005 (6)
C8	0.052 (9)	0.039 (9)	0.047 (9)	0.019 (7)	0.013 (7)	0.022 (7)
C7	0.031 (7)	0.046 (9)	0.044 (8)	0.009 (6)	0.003 (6)	−0.006 (7)
C3	0.045 (8)	0.040 (8)	0.038 (8)	0.009 (6)	−0.008 (7)	0.004 (6)
C4	0.049 (9)	0.079 (11)	0.016 (7)	−0.004 (8)	0.002 (6)	0.007 (7)
C12	0.039 (8)	0.045 (8)	0.027 (7)	0.013 (7)	−0.005 (6)	0.005 (6)
C24	0.040 (8)	0.019 (6)	0.044 (8)	0.014 (6)	−0.015 (6)	−0.002 (6)
C29	0.045 (8)	0.042 (9)	0.049 (9)	0.006 (7)	−0.002 (7)	0.002 (7)
C32	0.043 (8)	0.032 (8)	0.054 (9)	0.001 (6)	0.006 (7)	−0.007 (7)
C33	0.059 (10)	0.034 (8)	0.061 (10)	0.001 (7)	0.033 (8)	−0.003 (8)
C9	0.053 (9)	0.079 (12)	0.035 (8)	0.011 (9)	0.012 (7)	−0.005 (9)
C31	0.037 (8)	0.040 (8)	0.045 (8)	0.005 (6)	0.004 (6)	−0.005 (7)
C13	0.047 (8)	0.049 (9)	0.046 (9)	0.003 (7)	0.003 (7)	0.007 (7)
C5	0.040 (8)	0.053 (9)	0.035 (8)	0.003 (7)	−0.004 (6)	−0.011 (7)
C27	0.078 (12)	0.042 (9)	0.071 (12)	0.013 (9)	−0.042 (10)	0.001 (9)
C17	0.061 (10)	0.057 (10)	0.037 (8)	0.013 (8)	0.000 (7)	0.016 (7)
C25	0.039 (8)	0.039 (8)	0.080 (11)	0.017 (7)	−0.021 (8)	0.008 (8)
C28	0.084 (12)	0.070 (11)	0.027 (8)	0.017 (9)	−0.012 (8)	−0.007 (8)
C19	0.114 (14)	0.050 (10)	0.099 (13)	0.000 (10)	0.084 (12)	0.005 (9)
C26	0.046 (9)	0.064 (11)	0.091 (13)	0.001 (8)	−0.032 (9)	0.003 (10)
C14	0.023 (8)	0.092 (13)	0.077 (12)	0.022 (9)	0.002 (7)	0.016 (10)
C21	0.051 (10)	0.073 (12)	0.095 (13)	0.008 (9)	0.029 (9)	−0.003 (11)
C15	0.057 (10)	0.083 (12)	0.043 (9)	0.027 (9)	0.001 (8)	−0.012 (9)
C16	0.082 (12)	0.062 (11)	0.053 (10)	0.024 (9)	0.007 (9)	0.012 (8)
C20	0.20 (2)	0.055 (12)	0.123 (16)	0.059 (13)	0.114 (16)	0.036 (11)
C23	0.121 (15)	0.038 (10)	0.21 (2)	−0.015 (10)	0.127 (16)	−0.031 (12)
C22	0.108 (16)	0.054 (12)	0.22 (2)	−0.019 (11)	0.089 (17)	−0.064 (14)

### (II) (1,2-Diphenylpenta-1,3-dien-1-yl-5-ylidene)diiodido(triphenylphosphane)iridium(III) Geometric parameters (Å, º)

**Table:**

Ir1—C5	1.913 (12)	C24—C29	1.375 (16)
Ir1—C1	1.963 (11)	C24—C25	1.393 (15)
Ir1—P1	2.324 (3)	C29—C28	1.378 (16)
Ir1—I2	2.7061 (10)	C29—H29A	0.9500
Ir1—I1	2.7211 (10)	C32—C33	1.373 (16)
P1—C18	1.826 (11)	C32—C31	1.377 (15)
P1—C30	1.843 (11)	C32—H32A	0.9500
P1—C24	1.810 (11)	C33—H33A	0.9500
C11—C10	1.384 (15)	C9—H9A	0.9500
C11—C6	1.370 (16)	C31—H31A	0.9500
C11—H11A	0.9500	C13—C14	1.381 (17)
C1—C2	1.389 (15)	C13—H13A	0.9500
C1—C6	1.473 (15)	C5—H5A	0.9500
C10—C9	1.384 (17)	C27—C28	1.399 (19)
C10—H10A	0.9500	C27—C26	1.38 (2)
C30—C31	1.377 (15)	C27—H27A	0.9500
C30—C35	1.383 (14)	C17—C16	1.389 (17)
C34—C33	1.370 (17)	C17—H17A	0.9500
C34—C35	1.408 (15)	C25—C26	1.370 (17)
C34—H34A	0.9500	C25—H25A	0.9500
C6—C7	1.434 (15)	C28—H28A	0.9500
C35—H35A	0.9500	C19—C20	1.392 (18)
C18—C19	1.330 (16)	C19—H19A	0.9500
C18—C23	1.361 (17)	C26—H26A	0.9500
C2—C3	1.376 (15)	C14—C15	1.370 (19)
C2—C12	1.493 (15)	C14—H14A	0.9500
C8—C7	1.364 (16)	C21—C20	1.313 (19)
C8—C9	1.367 (17)	C21—C22	1.35 (2)
C8—H8A	0.9500	C21—H21A	0.9500
C7—H7A	0.9500	C15—C16	1.358 (18)
C3—C4	1.402 (16)	C15—H15A	0.9500
C3—H3A	0.9500	C16—H16A	0.9500
C4—C5	1.360 (15)	C20—H20A	0.9500
C4—H4A	0.9500	C23—C22	1.37 (2)
C12—C13	1.383 (16)	C23—H23A	0.9500
C12—C17	1.394 (16)	C22—H22A	0.9500
			
C5—Ir1—C1	89.5 (5)	C24—C29—C28	122.2 (13)
C5—Ir1—P1	89.2 (4)	C24—C29—H29A	118.9
C1—Ir1—P1	97.5 (3)	C28—C29—H29A	118.9
C5—Ir1—I2	92.6 (3)	C33—C32—C31	119.6 (13)
C1—Ir1—I2	84.2 (3)	C33—C32—H32A	120.2
P1—Ir1—I2	177.54 (8)	C31—C32—H32A	120.2
C5—Ir1—I1	113.6 (4)	C32—C33—C34	120.5 (12)
C1—Ir1—I1	156.0 (3)	C32—C33—H33A	119.7
P1—Ir1—I1	89.75 (8)	C34—C33—H33A	119.7
I2—Ir1—I1	88.01 (3)	C8—C9—C10	118.8 (12)
C18—P1—C30	103.4 (5)	C8—C9—H9A	120.6
C18—P1—C24	106.9 (6)	C10—C9—H9A	120.6
C30—P1—C24	102.1 (5)	C30—C31—C32	121.0 (12)
C18—P1—Ir1	115.2 (4)	C30—C31—H31A	119.5
C30—P1—Ir1	112.7 (4)	C32—C31—H31A	119.5
C24—P1—Ir1	115.1 (4)	C14—C13—C12	121.0 (13)
C10—C11—C6	122.0 (12)	C14—C13—H13A	119.5
C10—C11—H11A	119.0	C12—C13—H13A	119.5
C6—C11—H11A	119.0	C4—C5—Ir1	127.6 (10)
C2—C1—C6	123.6 (11)	C4—C5—H5A	116.2
C2—C1—Ir1	128.7 (9)	Ir1—C5—H5A	116.2
C6—C1—Ir1	107.6 (8)	C28—C27—C26	120.0 (13)
C11—C10—C9	119.9 (13)	C28—C27—H27A	120.0
C11—C10—H10A	120.0	C26—C27—H27A	120.0
C9—C10—H10A	120.0	C16—C17—C12	120.4 (13)
C31—C30—C35	119.7 (11)	C16—C17—H17A	119.8
C31—C30—P1	123.0 (9)	C12—C17—H17A	119.8
C35—C30—P1	117.2 (9)	C26—C25—C24	120.8 (14)
C33—C34—C35	120.1 (12)	C26—C25—H25A	119.6
C33—C34—H34A	120.0	C24—C25—H25A	119.6
C35—C34—H34A	120.0	C27—C28—C29	118.5 (14)
C11—C6—C1	123.3 (11)	C27—C28—H28A	120.8
C11—C6—C7	117.5 (11)	C29—C28—H28A	120.8
C1—C6—C7	118.9 (11)	C18—C19—C20	123.0 (15)
C30—C35—C34	119.0 (12)	C18—C19—H19A	118.5
C30—C35—H35A	120.5	C20—C19—H19A	118.5
C34—C35—H35A	120.5	C25—C26—C27	120.3 (14)
C19—C18—C23	115.4 (12)	C25—C26—H26A	119.9
C19—C18—P1	122.0 (10)	C27—C26—H26A	119.9
C23—C18—P1	122.6 (10)	C13—C14—C15	121.6 (14)
C3—C2—C1	121.0 (11)	C13—C14—H14A	119.2
C3—C2—C12	117.4 (11)	C15—C14—H14A	119.2
C1—C2—C12	121.7 (11)	C20—C21—C22	117.0 (16)
C7—C8—C9	122.5 (12)	C20—C21—H21A	121.5
C7—C8—H8A	118.7	C22—C21—H21A	121.5
C9—C8—H8A	118.7	C14—C15—C16	117.8 (14)
C8—C7—C6	119.2 (12)	C14—C15—H15A	121.1
C8—C7—H7A	120.4	C16—C15—H15A	121.1
C6—C7—H7A	120.4	C17—C16—C15	121.9 (15)
C2—C3—C4	126.0 (11)	C17—C16—H16A	119.1
C2—C3—H3A	117.0	C15—C16—H16A	119.1
C4—C3—H3A	117.0	C21—C20—C19	121.0 (17)
C5—C4—C3	123.4 (11)	C21—C20—H20A	119.5
C5—C4—H4A	118.3	C19—C20—H20A	119.5
C3—C4—H4A	118.3	C18—C23—C22	121.4 (14)
C13—C12—C17	117.1 (12)	C18—C23—H23A	119.3
C13—C12—C2	123.4 (12)	C22—C23—H23A	119.3
C17—C12—C2	119.4 (12)	C21—C22—C23	122.1 (15)
C29—C24—C25	118.3 (12)	C21—C22—H22A	119.0
C29—C24—P1	119.9 (9)	C23—C22—H22A	119.0
C25—C24—P1	121.7 (11)		
			
C6—C11—C10—C9	−2.3 (18)	C30—P1—C24—C29	84.2 (10)
C18—P1—C30—C31	−108.7 (10)	Ir1—P1—C24—C29	−38.2 (11)
C24—P1—C30—C31	2.2 (11)	C18—P1—C24—C25	16.5 (12)
Ir1—P1—C30—C31	126.3 (9)	C30—P1—C24—C25	−91.7 (11)
C18—P1—C30—C35	67.5 (10)	Ir1—P1—C24—C25	145.9 (9)
C24—P1—C30—C35	178.4 (9)	C25—C24—C29—C28	3.0 (19)
Ir1—P1—C30—C35	−57.5 (9)	P1—C24—C29—C28	−173.1 (10)
C10—C11—C6—C1	−171.6 (11)	C31—C32—C33—C34	1.0 (19)
C10—C11—C6—C7	1.9 (17)	C35—C34—C33—C32	−0.4 (19)
C2—C1—C6—C11	−60.9 (17)	C7—C8—C9—C10	1 (2)
Ir1—C1—C6—C11	118.0 (11)	C11—C10—C9—C8	0.7 (19)
C2—C1—C6—C7	125.8 (12)	C35—C30—C31—C32	3.1 (18)
Ir1—C1—C6—C7	−55.4 (12)	P1—C30—C31—C32	179.2 (9)
C31—C30—C35—C34	−2.5 (17)	C33—C32—C31—C30	−2.4 (18)
P1—C30—C35—C34	−178.8 (9)	C17—C12—C13—C14	0.5 (19)
C33—C34—C35—C30	1.2 (18)	C2—C12—C13—C14	−175.2 (12)
C30—P1—C18—C19	6.2 (14)	C3—C4—C5—Ir1	18.9 (19)
C24—P1—C18—C19	−101.1 (13)	C13—C12—C17—C16	0.4 (19)
Ir1—P1—C18—C19	129.5 (12)	C2—C12—C17—C16	176.3 (12)
C30—P1—C18—C23	−174.5 (14)	C29—C24—C25—C26	−3.8 (19)
C24—P1—C18—C23	78.2 (15)	P1—C24—C25—C26	172.3 (10)
Ir1—P1—C18—C23	−51.1 (15)	C26—C27—C28—C29	−2 (2)
C6—C1—C2—C3	174.1 (11)	C24—C29—C28—C27	0 (2)
Ir1—C1—C2—C3	−4.5 (17)	C23—C18—C19—C20	1 (3)
C6—C1—C2—C12	−5.7 (17)	P1—C18—C19—C20	−179.7 (15)
Ir1—C1—C2—C12	175.6 (8)	C24—C25—C26—C27	1 (2)
C9—C8—C7—C6	−1.5 (19)	C28—C27—C26—C25	2 (2)
C11—C6—C7—C8	0.0 (17)	C12—C13—C14—C15	0 (2)
C1—C6—C7—C8	173.8 (11)	C13—C14—C15—C16	−1 (2)
C1—C2—C3—C4	−6.4 (19)	C12—C17—C16—C15	−2 (2)
C12—C2—C3—C4	173.5 (12)	C14—C15—C16—C17	2 (2)
C2—C3—C4—C5	−1 (2)	C22—C21—C20—C19	−3 (3)
C3—C2—C12—C13	129.0 (13)	C18—C19—C20—C21	0 (3)
C1—C2—C12—C13	−51.1 (17)	C19—C18—C23—C22	1 (3)
C3—C2—C12—C17	−46.6 (16)	P1—C18—C23—C22	−178.0 (17)
C1—C2—C12—C17	133.3 (12)	C20—C21—C22—C23	6 (3)
C18—P1—C24—C29	−167.6 (9)	C18—C23—C22—C21	−5 (4)

### (II) (1,2-Diphenylpenta-1,3-dien-1-yl-5-ylidene)diiodido(triphenylphosphane)iridium(III) Hydrogen-bond geometry (Å, º)

**Table:**

*D*—H···*A*	*D*—H	H···*A*	*D*···*A*	*D*—H···*A*
C7—H7*A*···I2	0.95	2.94	3.707 (12)	138
C23—H23*A*···I1	0.95	2.84	3.699 (17)	152
